# A structural equation modeling approach to examine determinants of nutritional status in Palestinian children 6–59 months

**DOI:** 10.1371/journal.pone.0331579

**Published:** 2025-09-08

**Authors:** Ghada Issa, Radwan Qasrawi, Suliman Thwib, Malak Amro, Razan Abu Ghoush, Sabri Saghir, Doa’a Mujahed, Maysaa Nemer, Mousa Halaika, Manal Badrasawi, Ayoub Al-Jawaldeh, Ibrahim Elmadfa, Lara Nasreddine, Diala Abu Al-Halawa, Maisan Nimer

**Affiliations:** 1 Department of Computer Sciences, Al Quds University, Jerusalem, Palestine; 2 Department of Computer Engineering, Istinye University, Istanbul, Turkey; 3 The Center of Technology and Innovation, Al-Quds University, Jerusalem, Palestine; 4 Department of Nutrition and Food technology, College of Agriculture, Hebron University, Hebron, Palestine; 5 Institute Of Community and Public Health, Birzeit university, Ram Allah, Palestine; 6 Nutrition Department Director, Ministry of Health, Palestine; 7 Nutrition and Food Technology Department, Faculty of Agriculture and Vetrenary Medicine, An-Najah National University, Palestine; 8 Regional Office for the Eastern Mediterranean, World Health Organization, Cairo, Egypt; 9 Department of Nutrition, Faculty of Life Sciences, University of Vienna, Vienna, Austria; 10 Nutrition and Food Sciences Department, Faculty of Agriculture and Food Sciences, American University of Beirut, Beirut, Lebanon; 11 Department of Medicine, Al-Quds University, Jerusalem, Palestine; Universidade de Sao Paulo Faculdade de Saude Publica, BRAZIL

## Abstract

**Background:**

Undernutrition remains a persistent public health concern among young children in Palestine, shaped by a range of socioeconomic and dietary factors. This study applies a Structural Equation Modeling (SEM) approach to explore both direct and indirect determinants of child growth among children aged 6–59 months in the West Bank.

**Methods:**

Data were drawn from a 2022 cross-sectional survey involving 300 children selected from 1,400 households. Child growth was assessed using anthropometric indicators (HAZ, WAZ, MUAC) and dietary adequacy via the Mean Nutrient Inadequacy Ratio (MNIR). Latent constructs were developed for socioeconomic status, food insecurity, dietary intake (macro- and micronutrients), feeding practices, neonatal health, and food availability. SEM was used to examine interrelationships and the pathways influencing child nutritional status.

**Results:**

Descriptive analysis showed substantial variation in undernutrition and nutrient intake across regions, family size, and parental education. SEM results showed that macronutrient intake had the strongest direct effect on child growth, followed by socioeconomic status and neonatal health. Infant feeding practices and micronutrient intake also contributed positively, though their effects were primarily indirect. Food availability acted as a significant mediator, linking structural access issues to poor dietary diversity and feeding behavior. The model demonstrated good fit across multiple indices.

**Conclusion:**

Child nutrition in Palestine is shaped by interrelated determinants of dietary intake, caregiver practices, food access, and early-life health conditions. Effective interventions must target both nutritional quality and the broader social and environmental context to improve growth outcomes. SEM offers a powerful framework to understand these pathways and guide evidence-based policy design.

## 1. Introduction

Healthy growth during childhood is widely seen as a key sign of overall well-being. It’s influenced by a mix of biological, environmental, nutritional, and socioeconomic factors [1,2]. Since these factors are closely linked, Structural Equation Modeling (SEM) is a useful tool for studying how they interact and affect children’s health. SEM allows researchers to look at both direct and indirect effects at the same time, offering a clearer picture of how different elements work together. Unlike traditional methods that often analyze one relationship at a time, SEM connects the associations between variables. This makes it especially valuable for understanding how things like food insecurity, eating habits, and parenting practices impact children’s growth.

Several studies have demonstrated that limited food access and poor dietary quality are linked to stunting and other growth impairments. For instance, Dukhi et al. conducted an SEM-based study in Pakistan and found that food insecurity directly contributed to malnutrition among children under five years old, with indirect effects mediated through parental education and income levels [3]. Meitei applied SEM to analyze the relationship between poverty, food insecurity, and child mortality in India, demonstrating that improving economic conditions and access to healthcare significantly reduced undernutrition rates [4]. These findings showed the power of SEM in identifying both the immediate and mediated effects of socioeconomic and nutritional factors on child health.

Healthy childhood growth is critical not only for physical health, but also for cognitive development [5]. Disturbances in these factors may lead to various outcomes such as stunting, poor cognitive performance, and, in turn, mortality and morbidity [6]. Furthermore, unhealthy nutritional status, particularly a lack in key vitamins and minerals, has repeatedly been shown to be a root cause of these developmental abnormalities, showing the necessity of adequate and balanced dietary consumption during critical times of childhood growth [6,7].

Access to healthcare, food, or even clean water can vary widely across different geographic regions. Socioeconomic and regional disparities are important determinants of healthy child growth. In resource-limited environments, children under five are particularly vulnerable because they depend entirely on others for nutritional provision and health requirements [8,9]. Research consistently shows a strong association between food insecurity and suboptimal growth outcomes, which emphasizes the need for detailed approaches that address the root causes of malnutrition [10–13]. Moreover, malnutrition and food insecurity are especially severe in regions experiencing economic poverty, and social disruption. Children in these environments are particularly vulnerable to undernutrition, which commonly presents as stunting or micronutrients deficiencies [14,15]. These disparities are apparent in Palestine where the current political situation and the socio-political implications thereof have prevented timely and more efficient access to basic needs and services, such as food, health care, and education [16]. Data indicate drastic inequalities in child health and nutrition among urban, rural, and refugee populations in Palestine. Refugee children, for instance, show increased rates of stunting and other growth deficits compared with children living in urban and rural areas. This disparity is likely due to variations in living conditions, allocation of resources, and access to healthcare and sanitation [16,17]. Furthermore, studies showed that higher parental education directly contributes to improved nutritional status and indirectly affects child growth by promoting healthier household practices and better resource allocation [18,19]. Recent reviews further highlight that in regions facing economic and political challenges, these effects are even more important [20].

Despite extensive research on child growth determinants, the integrated influence of food insecurity, nutrition, and socioeconomic factors on child development in Palestine remains poorly understood. Facing economic hardship and political instability, the region experiences heightened food insecurity and limited access to essential nutrition. Previous studies have explored these factors individually; however, an integrated analysis is missing. This study fills that gap by applying Structural Equation Modeling (SEM) to an extensive dataset of Palestinian children under five. It quantitatively examines how socioeconomic status, household food security, and dietary practices interact to affect growth outcomes, building on existing literature linking food insecurity and regional disparities to child development. Furthermore, the research aims to guide practical interventions to reduce malnutrition and promote equitable growth in Palestine and other conflict-affected regions.

## 2. Methodology

This study employs Structural Equation Modeling (SEM) to investigate both the direct and indirect determinants of growth among Palestinian children aged 6 months to 59 months (5 years). The primary focus is on exploring the impact of socioeconomic conditions, household food insecurity, nutritional practices, and dietary intake on child growth outcomes.

### 2.1 Data collection and sampling

The analysis was conducted based on data from a 2022 cross-sectional survey conducted in the West Bank, Palestine [21], which assessed food insecurity across 1,400 households. Questionnaires were distributed starting December 5, 2022, and data collection concluded on April 16, 2023. From this sample, a subset of 300 households with children aged 6–59 months was selected for further analysis. All participants had provided written informed consent prior to participation. The data, accessed on February 24, 2025, contained no identifiable personal information. The study adhered to the principles of the Declaration of Helsinki and was approved by the Institutional Review Board at Hebron University (Approval Code: IRB 17/7, Date of Approval: 17 October 2022).

### 2.3 Study variables

A face-to-face questionnaire was administered to capture multiple variables as outlined in Table 1.

**Table 1:** Study variables.

Construct	Description
Child Growth	Assesses the nutritional and physical development of children aged 6–59 months, using WHO-standardized anthropometric indicators including Weight-for-Age, Height-for-Age, MUAC, BMI, and waist circumference [22]. Also includes the Mean Nutrient Inadequacy Ratio to reflect dietary sufficiency across essential nutrients [23].
Socioeconomic and Demographic	Captures household background variables such as child’s age and gender, household income, family size, parental education, employment status, and refugee status.
Household Food Insecurity	Measured using the Radimer/Cornell Hunger Scale, which assesses food insecurity through four dimensions: food quantity, food quality, acceptability of food sources, and certainty of access to food [24].
Dietary Intake	Based on two 24-hour dietary recalls (weekday and weekend), detailing types of food and beverages consumed, meal timing, preparation methods, and macro- and micronutrient intake. This reflects the child’s actual dietary behavior and nutrient intake [21].
Nutrition-related Practices	Describes household food behaviors such as dietary routines, meal patterns, food purchasing and preparation habits, and decision-making autonomy in food-related matters.
Neonatal Health	Comprises early-life biological indicators including birth weight and gestational age, which are critical predictors of infant growth and long-term nutritional outcomes.
Infant Feeding Practices	Includes attitudes and knowledge related to food diversity, appropriate timing for food introduction, and feeding frequency during infancy.
Food Availability	Captures barriers to household food access, including difficulties in achieving dietary diversity, food access frequency, and challenges in maintaining extended breastfeeding. Reflects structural food security constraints that affect child nutrition.

#### 2.3.1 Nutritional Status Assessment (dietary intake).

We collected dietary intake data using three 24-hour recalls on non-consecutive days over a six-month period, including a weekend day, to better reflect day-to-day and seasonal variation in consumption. To improve accuracy and minimize missing information, trained interviewers used the multiple-pass method, which begins with an open-ended recall followed by a structured list-based approach to prompt recall of forgotten foods. Portion sizes were estimated using the local food atlas and household tools.

The collected dietary data were analyzed with Version 1 of the Eastern Mediterranean Food Intake Database (EMFID) software. Developed by Al-Quds University in partnership with the World Health Organization (WHO) in 2021, EMFID provides food-composition data tailored to the Eastern Mediterranean region. Using this software, the reported foods and beverages were converted into detailed nutrient values, including macronutrients (such as energy, protein, fats, carbohydrates, and fiber) and micronutrients (including B vitamins, vitamin C, vitamin A, calcium, magnesium, potassium, phosphorus, copper, iron, and zinc). The daily nutrient intake for each child was calculated by averaging the values from the three recorded days, in order to improve accuracy.

The nutrient intake data were then assessed in comparison with the Recommended Dietary Allowances (RDAs) established by the U.S. National Research Council [25]. These RDAs serve as standard reference values, adjusted by age, gender, and anthropometric characteristics, to determine whether a child’s intake meets, exceeds, or falls short of recommended nutritional levels.

#### 2.3.2 Anthropometric measurements.

Children’s physical growth was assessed through a set of anthropometric measurements, including height, weight, and mid-upper arm circumference (MUAC). Height was measured using a portable SECA 217 stadiometer with a headboard, and each child’s height was recorded twice to ensure precision to the nearest 0.1 cm. Weight was measured with a SECA 874 digital scale (accurate to 0.1 kg), with children asked to remove shoes, socks, and heavy clothing beforehand.

These measurements were processed using WHO Anthro Software (Version 3, 2009) to compute standardized growth indices: height-for-age Z-score (HAZ), weight-for-age Z-score (WAZ), and weight-for-height Z-score (WHZ) [22]. These indices allowed classification of children under five years into categories of underweight, stunting, and wasting, with moderate levels defined by Z-scores below −2 and severe levels below −3.

MUAC measurements, taken specifically for older children, were conducted using a NutriActiva MUAC tape [21]. The tape was placed at the midpoint between the shoulder and elbow of the left upper arm, and each child’s MUAC was measured twice to the nearest 0.1 cm, with the average recorded as the final value. MUAC-for-age Z-scores (MUACZ) were also calculated using WHO Anthro Software, and children were classified as moderately undernourished if their MUACZ scores fell below −2 or severely undernourished if they fell below −3.

### 2.4 Structural Equation Modeling (SEM)

This study employed Structural Equation Modeling (SEM) to examine the relationships among key determinants of child growth [26]. The analysis started with descriptive statistics to summarize the characteristics of the study population. Measures of central tendency and dispersion, such as means and standard deviations, were reported alongside frequencies and percentages for categorical variables. Univariate correlations were then calculated to explore the pairwise associations among growth indicators and nutritional variables, serving as a preliminary step to identify relevant relationships for inclusion in the model.

Confirmatory Factor Analysis (CFA) was conducted in AMOS version 20 to validate the measurement model. The latent-construct adequacy was assessed by confirming that each observed variable loaded at ≥ 0.50 (p < .05) on its intended factor. Convergent validity with Average Variance Extracted (AVE) > 0.50 and internal consistency with Composite Reliability (CR) > 0.60 [27]. Model fit was evaluated using multiple indices: The Chi-square (χ²) statistic, Normed Fit Index (NFI), Comparative Fit Index (CFI), and Root Mean Square Error of Approximation (RMSEA). A satisfactory model fit was indicated by a non-significant χ², NFI and CFI values of 0.950 or higher, and RMSEA values not exceeding 0.070 [28]. Parameters were estimated primarily using the Maximum Likelihood (ML) method. In cases where multivariate normality was violated, Bayesian estimation procedures were applied to enhance robustness.

Upon confirmation of the measurement model, the structural model was estimated using AMOS version 20. The structural model tested direct and indirect paths from socioeconomic status, food insecurity, dietary intake, and parental nutritional practices to growth outcomes (HAZ, WAZ, WHZ, and MUACZ). This framework allowed for the examination of mediating pathways linking social and dietary factors to physical growth.

## 3.Results

The analysis started with descriptive statistics, focusing on key anthropometric indicators (HAZ, WAZ, MUAC) and dietary adequacy, as measured by the Mean Nutrient Inadequacy Ratio (MNIR), across various socioeconomic and demographic subgroups. The SEM analysis further explores the direct and indirect relationships among socioeconomic conditions, household food insecurity, dietary intake, neonatal and infant feeding practices, nutrition-related behaviors, food availability, and their combined effects on child growth outcomes.

### 3.1 Descriptive analysis

The results in Table 2 show that the prevalence of stunting, wasting, and abnormal MUAC among Palestinian children aged 6–59 months varies across socioeconomic and demographic groups. Children living in refugee camps had the highest stunting rate (39%), followed by those in villages (23.4%) and cities (22.8%). Wasting and abnormal MUAC were also slightly more common in camps, indicating greater nutritional risk in these settings. By district, stunting was most prevalent in the Middle region (32.9%), while the North had the highest rate of abnormal MUAC (27.6%). Household income showed minimal variation in undernutrition rates, with slightly higher stunting and abnormal MUAC observed in higher-income groups, indicating the influence of factors beyond income, such as feeding practices or parental knowledge. Furthermore, larger family size was associated with poorer nutritional outcomes. Households with seven or more members reported the highest stunting (35.9%) and wasting (15.4%) rates, while no cases of wasting or abnormal MUAC were reported among families with one or two members. Significantly higher rates of stunting (46.6%) and wasting (22.4%) were observed among children whose caregivers had only secondary education or less, compared to those with university-educated parents, highlighting parental education as a key factor.

**Table 2:** Distribution of anthropometric status (wasting, stunting, and MUAC) by socioeconomic and demographic factors among palestinian children aged 1-5 years.

	Wasting n(%)		Stunting n(%)		MUAC n(%)	
	No	Yes	No	Yes	Normal	Abnormal
**Locality**						
City	142(89.9)	16(10.1)	122(77.2)	36(22.8)	120(75.9)	38(24.1)
Village	98(88.3)	13(11.7)	85(76.6)	26(23.4)	86(77.5)	25(22.5)
Camp	36(87.8)	5(12.2)	25(61)	16(39)	32(78)	9(22)
**District**						
South	119(89.5)	14(10.5)	98(73.7)	35(26.3)	98(73.7)	35(26.3)
Middle	72(91.1)	7(8.9)	53(67.1)	26(32.9)	69(87.3)	10(12.7)
North	85(86.7)	13(13.3)	81(82.7)	17(17.3)	71(72.4)	27(27.6)
**Total Income**						
Low	25(89.3)	3(10.7)	21(75)	7(25)	22(78.6)	6(21.4)
Moderate	195(89.4)	23(10.6)	164(75.2)	54(24.8)	166(76.1)	52(23.9)
High	56(87.5)	8(12.5)	47(73.4)	17(26.6)	50(78.1)	14(21.9)
**Family Size**						
1 −2	7(100)	0(0)	6(85.7)	1(14.3)	7(100)	0(0)
3 −4	148(89.2)	18(10.8)	121(72.9)	45(27.1)	124(74.7)	42(25.3)
5 −6	88(89.8)	10(10.2)	80(81.6)	18(18.4)	75(76.5)	23(23.5)
7+	33(84.6)	6(15.4)	25(64.1)	14(35.9)	32(82.1)	7(17.9)
**Household Education**						
<=Secondary	45(77.6)	13(22.4)	31(53.4)	27(46.6)	46(79.3)	12(20.7)
University	231(91.7)	21(8.3)	201(79.8)	51(20.2)	192(76.2)	60(23.8)

The results presented in Table 3 show considerable variation in vitamin intake among Palestinian children aged 6–59 months, with notable differences across both sex and age groups. Vitamin A deficiency was the most prevalent, affecting 86.8% of the children, with slightly higher rates among boys than girls. The highest proportion of inadequate intake was observed in children aged 48–60 months (95.9%), indicating a potential deterioration in vitamin A consumption with age. Vitamin B1 intake was also less than the recommended standards, with about half of the children (51.6%) falling below adequate levels, and higher inadequacy rates among younger age groups. In contrast, intake of vitamins B2, B5, and B6 was relatively better, with over two-thirds of children meeting recommended levels. However, older children (48–60 months) again showed poorer intake in some cases, particularly for vitamin B5.

**Table 3:** Distribution of vitamins intake among palestinian children aged 1-5 years.

Vitamins	Sex n(%)		Age n(%)					
	Boys	Girls	6-12	12-24	24-36	36-48	48-60	Total
Vit A								
Inadequate	136(88.9)	133(84.7)	52(83.9)	61(80.3)	63(92.6)	46(83.6)	47(95.9)	269(86.8)
Adequate	17(11.1)	24(15.3)	10(16.1)	15(19.7)	5(7.4)	9(16.4)	2(4.1)	41(13.2)
Vit B1								
Inadequate	81(52.9)	79(50.3)	39(62.9)	41(53.9)	31(45.6)	22(40)	27(55.1)	160(51.6)
Adequate	72(47.1)	78(49.7)	23(37.1)	35(46.1)	37(54.4)	33(60)	22(44.9)	150(48.4)
Vit B2								
Inadequate	53(34.6)	52(33.1)	20(32.3)	21(27.6)	21(30.9)	19(34.5)	24(49)	105(33.9)
Adequate	100(65.4)	105(66.9)	42(67.7)	55(72.4)	47(69.1)	36(65.5)	25(51)	205(66.1)
Vit B3								
Inadequate	86(56.2)	87(55.4)	42(67.7)	44(57.9)	28(41.2)	26(47.3)	33(67.3)	173(55.8)
Adequate	67(43.8)	70(44.6)	20(32.3)	32(42.1)	40(58.8)	29(52.7)	16(32.7)	137(44.2)
Vit B5								
Inadequate	44(28.8)	44(28)	19(30.6)	14(18.4)	13(19.1)	14(25.5)	28(57.1)	88(28.4)
Adequate	109(71.2)	113(72)	43(69.4)	62(81.6)	55(80.9)	41(74.5)	21(42.9)	222(71.6)
Vit B6								
Inadequate	34(22.2)	36(22.9)	21(33.9)	16(21.1)	12(17.6)	9(16.4)	12(24.5)	70(22.6)
Adequate	119(77.8)	121(77.1)	41(66.1)	60(78.9)	56(82.4)	46(83.6)	37(75.5)	240(77.4)
Vit B12								
Inadequate	57(37.3)	49(31.2)	23(37.1)	24(31.6)	20(29.4)	18(32.7)	21(42.9)	106(34.2)
Adequate	96(62.7)	108(68.8)	39(62.9)	52(68.4)	48(70.6)	37(67.3)	28(57.1)	204(65.8)
Folate								
Inadequate	94(61.4)	93(59.2)	41(66.1)	47(61.8)	41(60.3)	26(47.3)	32(65.3)	187(60.3)
Adequate	59(38.6)	64(40.8)	21(33.9)	29(38.2)	27(39.7)	29(52.7)	17(34.7)	123(39.7)
Vit C								
Inadequate	52(34)	54(34.4)	41(66.1)	24(31.6)	10(14.7)	14(25.5)	17(34.7)	106(34.2)
Adequate	101(66)	103(65.6)	21(33.9)	52(68.4)	58(85.3)	41(74.5)	32(65.3)	204(65.8)

For vitamin B3 and folate, more than half of the children had inadequate intake (55.8% and 60.3%, respectively), with minimal differences between boys and girls. Children aged 6–12 months and 48–60 months reported the lowest adequacy levels. Vitamin B12 and vitamin C showed more favorable results, with around two-thirds of children meeting adequate intake. Nevertheless, a significant set of children aged 6–12 months remained at risk of deficiency, particularly for vitamin C, where 66.1% were below adequate intake.

The results in Table 4 show varying levels of mineral intake adequacy among Palestinian children aged 6–59 months, with several deficiencies more important by age than by sex. Calcium inadequacy was among the highest levels, affecting 69% of the children, with the highest rates among those aged 48–60 months (95.9%). Girls had slightly better intake levels than boys, though inadequacy remained high across both sexes. Magnesium intake was more adequate overall, with 68.7% of children meeting recommended levels. However, adequacy dropped notably in the 48–60-month group (46.9%). For manganese, 42.6% of children had inadequate intake, with younger children (6–12 months) showing the highest adequacy (80.6%). In contrast, older children aged 24–36 and 48–60 months showed higher inadequacy rates.

**Table 4:** Distribution of minerals intake among Palestinian children aged 1-5 years.

Minerals	Sex n(%)		Age n(%)					
	Boys	Girls	6-12	12-24	24-36	36-48	48-60	Total
Calcium								
Inadequate	109(71.2)	105(66.9)	16(25.8)	55(72.4)	53(77.9)	43(78.2)	47(95.9)	214(69)
Adequate	44(28.8)	52(33.1)	46(74.2)	21(27.6)	15(22.1)	12(21.8)	2(4.1)	96(31)
Mg								
Inadequate	49(32)	48(30.6)	24(38.7)	20(26.3)	13(19.1)	14(25.5)	26(53.1)	97(31.3)
Adequate	104(68)	109(69.4)	38(61.3)	56(73.7)	55(80.9)	41(74.5)	23(46.9)	213(68.7)
Manganese								
Inadequate	66(43.1)	66(42)	12(19.4)	34(44.7)	38(55.9)	20(36.4)	28(57.1)	132(42.6)
Adequate	87(56.9)	91(58)	50(80.6)	42(55.3)	30(44.1)	35(63.6)	21(42.9)	178(57.4)
Iron								
Inadequate	74(48.4)	93(59.2)	33(53.2)	36(47.4)	41(60.3)	21(38.2)	36(73.5)	167(53.9)
Adequate	79(51.6)	64(40.8)	29(46.8)	40(52.6)	27(39.7)	34(61.8)	13(26.5)	143(46.1)
Zinc								
Inadequate	58(37.9)	56(35.7)	36(58.1)	21(27.6)	15(22.1)	13(23.6)	29(59.2)	114(36.8)
Adequate	95(62.1)	101(64.3)	26(41.9)	55(72.4)	53(77.9)	42(76.4)	20(40.8)	196(63.2)

Iron deficiency was observed in over half the children (53.9%), with higher rates among girls (59.2%) compared to boys (48.4%). The 48–60-month group again showed the highest inadequacy (73.5%). Regarding zinc, 36.8% of children had inadequate intake, with the youngest (6–12 months) and oldest (48–60 months) age groups having the poorest intake (58.1% and 59.2%, respectively). Children aged 24–48 months had the highest adequacy.

The findings from Table 5 indicate variable adequacy in macronutrient intake among Palestinian children aged 6–59 months, with differences evident by age and sex. Carbohydrate intake was inadequate in 36.1% of children, with slightly higher inadequacy among girls (38.2%) compared to boys (34%). The highest deficiency was seen in the 6–12-month group (56.5%), while intake improved progressively with age, reaching 71.4% adequacy in the 48–60-month group. Protein intake was largely sufficient across the sample, with 87.7% of children meeting recommended levels. However, children aged 6–12 months showed the lowest adequacy (56.5%), indicating a higher vulnerability to protein deficiency during infancy. Adequacy improved significantly with age, with over 90% of children older than 24 months meeting the recommended protein intake. The Fat intake showed the highest overall inadequacy among the three macronutrients, with 40% of children below the recommended level. Again, the 6–12-month age group had the most important inadequacy (82.3%). As age increased, fat intake adequacy also improved, reaching over 73% in the 36–60-month range.

**Table 5:** Distribution of macronutrient intake among Palestinian children aged 1-5 years.

Minerals	Sex n(%)		Age n(%)					
	Boys	Girls	6-12	12-24	24-36	36-48	48-60	Total
Carb								
Inadequate	52(34)	60(38.2)	35(56.5)	25(32.9)	22(32.4)	16(29.1)	14(28.6)	112(36.1)
Adequate	101(66)	97(61.8)	27(43.5)	51(67.1)	46(67.6)	39(70.9)	35(71.4)	198(63.9)
Protein								
Inadequate	17(11.1)	21(13.4)	27(43.5)	5(6.6)	1(1.5)	1(1.8)	4(8.2)	38(12.3)
Adequate	136(88.9)	136(86.6)	35(56.5)	71(93.4)	67(98.5)	54(98.2)	45(91.8)	272(87.7)
Fat								
Inadequate	59(38.6)	65(41.4)	51(82.3)	27(35.5)	18(26.5)	15(27.3)	13(26.5)	124(40)
Adequate	94(61.4)	92(58.6)	11(17.7)	49(64.5)	50(73.5)	40(72.7)	36(73.5)	186(60)

### 3.2 SEM analysis

#### 3.2.1 Model fit.

The results presented in Table 6 demonstrate that the structural equation model (SEM) assessing the determinants of child growth among Palestinian children aged 6–59 months achieved an overall acceptable to good model fit.

**Table 6:** Goodness-of-fit indices for the Structural Equation Model (SEM) of the determinants of child growth among Palestinian children aged 1- 5 years.

Fit Index	Value	Interpretation
χ²/df	(652.014/421) 1.549	Good fit: *< 3.0 indicates good fit*
CFI	0.857	Acceptable fit: *≥ 0.80 is acceptable, ≥ 0.90 is good*
TLI	0.842	Acceptable fit: *≥ 0.80 is acceptable, ≥ 0.90 is good*
RMSEA	0.042	Good fit: *< 0.05 good, < 0.08 reasonable*
RMSEA 90% CI	[0.036, 0.048]	Good fit: *upper bound < 0.08 indicates good precision*
PCLOSE	0..982	Good fit: *> 0.05 suggests good fit*
SRMR/RMR	0.013	Excellent fit: *< 0.05 excellent, < 0.08 good*
GFI	0.882	Acceptable fit: *≥ 0.80 is acceptable, ≥ 0.90 is good*
AGFI	0.861	Acceptable fit: *≥ 0.80 is acceptable*

The Chi-square to degrees of freedom ratio (χ²/df = 1.549) falls well below the commonly accepted threshold of 3.0, indicating a good fit between the observed and estimated models. Similarly, the Root Mean Square Error of Approximation (RMSEA = 0.042) is well within the threshold for a good fit (<0.05), with its 90% confidence interval [0.036, 0.048] also remaining below 0.08, suggesting high precision in the model estimates. The PCLOSE value (0.982) further supports the model’s adequacy, indicating that the RMSEA is not significantly greater than 0.05.

The Standardized Root Mean Square Residual (SRMR = 0.013) also indicates an excellent fit, being far below the 0.05 threshold. For incremental fit indices, both the Comparative Fit Index (CFI = 0.857) and Tucker-Lewis Index (TLI = 0.842) fall within the acceptable range (≥ 0.80), though slightly below the conventional cutoff for a “good” fit (≥ 0.90). The absolute fit indices such as the Goodness-of-Fit Index (GFI = 0.882) and Adjusted Goodness-of-Fit Index (AGFI = 0.861) also fall within acceptable limits, further confirming that the model sufficiently captures the original structure of the data.

#### 3.2.2 SEM diagram.

**Fig 1:**
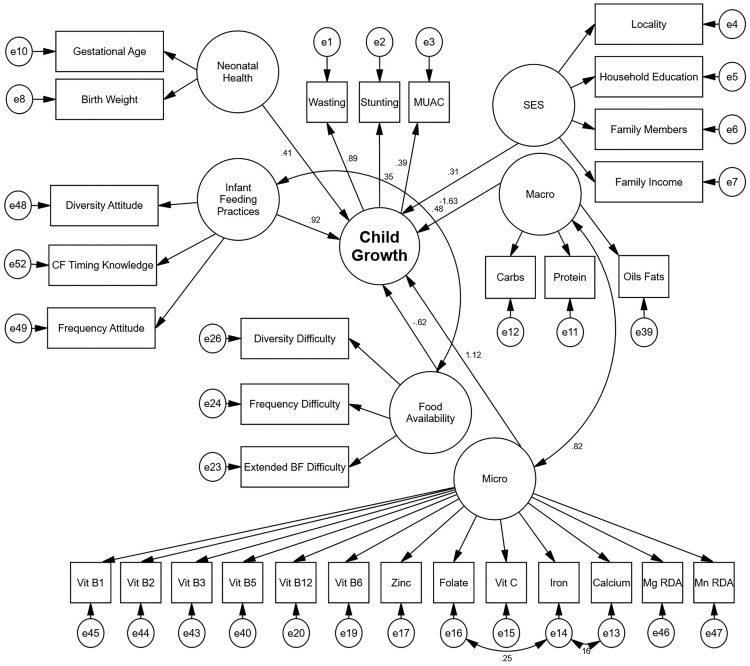
Structural Equation Model of determinants of child growth among Palestinian children aged 6-59 months.

Fig 1 presents the structural equation model outlining the pathways influencing child growth among Palestinian children aged 6–59 months. In this model, Child Growth is represented as a latent construct measured by three observed indicators: wasting, stunting, and MUAC (Mid-Upper Arm Circumference) Z-scores, which together reflect both acute and chronic undernutrition.

The model illustrates several key determinants and their interrelationships. Socioeconomic and Demographic Status (SES), comprised of locality, household education, family size, and income, has a direct effect on child growth (path coefficient = 0.31), highlighting the foundational role of household resources and social conditions in influencing nutritional outcomes.

Food Availability, which includes reported difficulties in dietary diversity, meal frequency, and extended breastfeeding, shows a strong negative direct association with child growth (path coefficient = −0.62), indicating that limited access to consistent and diverse foods is associated with poorer growth outcomes.

Child growth is positively influenced by Micronutrient Intake (Mirco) (path coefficient = 1.12), which itself is composed of multiple observed indicators including vitamins (B1, B2, B3, B5, B6, B12, C, folate) and minerals (zinc, iron, calcium, magnesium, manganese). It is also influenced by Macronutrient Intake (Macro) (path coefficient = 1.63), reflected by intake of carbohydrates, proteins, and fats. Macronutrient Intake is positively correlated with Micronutrient Intake (correlation coefficient = 0.82), indicating interconnected dietary patterns.

Furthermore, Infant Feeding Practices, including capturing attitudes and knowledge regarding food diversity, complementary feeding timing, and feeding frequency, demonstrate a strong direct effect on child growth (path coefficient = 0.92). Neonatal Health, based on gestational age and birth weight, also contributes positively (path coefficient = 0.41), emphasizing the importance of early-life conditions.

#### 3.2.3 Latent-indicator standardized direct effects.

The structural equation model results reveal several statistically significant direct effects on child growth among Palestinian children aged 6–59 months. Table 7 shows that macronutrient intake has the strongest direct effect (β = 1.628, *p* = .004), highlighting the critical role of energy and protein sources in supporting physical development. Socioeconomic status (SES) also demonstrates a significant positive association with child growth (β = 0.309, *p* = .005), reinforcing the influence of household resources and education on child nutrition. Neonatal health (β = 0.409, *p* = .039) significantly contributes to growth outcomes, indicating that better birth conditions (e.g., higher birth weight and gestational age) positively affect early development. Micronutrient intake (β = 1.123) and feeding practices (β = 0.922) exhibit strong effect sizes, though their associations did not reach conventional statistical significance (*p* = .067 and.099, respectively).

**Table 7:** Standardized direct effects and statistical significance in the structural equation model of child growth determinants.

Causal Variable	Effect variable < --	Direct Effect	Significance (P-Value)
**Child Growth**	SES	.309	.005
	Food Availability	.623	.063
	Macronutrients	1.628	.004
	Micronutrients	1.123	.067
	Neonatal Health	.409	.039
	Feeding Practices	.922	.099
	Wasting	.885	<.001
	Stunting	.352	<.001
	MUAC	.391	<.001
**SES**	Locality	.264	<.001
	Total Income	.149	.019
	Family Size	. 149	.019
	Household Education	1.014	.067
**Food Availability**	Diversity Difficulty	.653	<.001
	Frequency Difficulty	.796	<.001
	Extended Breastfeeding Difficulty	.191	.005
**Macronutrients**	Protein	.617	<.001
	Carb	.724	<.001
	Fat	.225	<.001
**Micronutrients**	Vitamin C	.358	.005
	Vitamin B1	.588	.002
	Vitamin B2	.520	.003
	Vitamin B3	.596	.002
	Vitamin B5	.478	.003
	Vitamin B6	.622	.002
	Vitamin B12	.518	.003
	Folate	.546	.002
	Iron	.553	.003
	Zinc	.727	.002
	Calcium	.190	<.001
	Magnesium	.728	.002
	Manganese	.367	.005
**Neonatal Health**	Gestational Age	.361	.034
	Birth Weight	.446	<.001
**Feeding Practices**	Diversity Attitude	.282	<.001
	Feed Timing Knowledge	.010	.912
	Frequency Attitude	.149	.147

In terms of anthropometric outcomes, wasting, stunting, and MUAC all significantly reflect the latent construct of child growth, with strong standardized loadings (β = 0.885, 0.352, and 0.391, respectively; all *p* < .001), confirming their reliability as indicators.

The construct of SES is significantly shaped by key variables, including locality (β = 0.264, *p* < .001), household income, and family size (both β = 0.149, *p* = .019). Household education also contributes notably (β = 1.014), though its effect is marginally significant (*p* = .067). Food availability is significantly influenced by challenges in food diversity (β = 0.653), frequency of access (β = 0.796), and extended breastfeeding (β = 0.191), with all effects statistically significant (*p* < .01), highlighting how structural barriers in household food access limit dietary sufficiency.

Among macronutrients, carbohydrates (β = 0.724), protein (β = 0.617), and fat (β = 0.225) all significantly load onto the latent construct, indicating balanced contributions from all three in shaping dietary energy intake (*p* < .001 for all). Regarding micronutrients, all vitamins and minerals exhibit statistically significant loadings (*p* < .01), with the strongest effects observed for magnesium (β = 0.728), zinc (β = 0.727), and vitamin B1 (β = 0.588). Within neonatal health, both gestational age (β = 0.361, *p* = .034) and birth weight (β = 0.446, *p* < .001) are significant contributors, supporting early-life factors as strong predictors of future growth. For feeding practices, a positive attitude toward food diversity significantly contributes (β = 0.282, *p* < .001), while knowledge of feeding timing and feeding frequency attitude show non-significant associations, indicating that behavioral orientation may be more influential than factual knowledge alone in shaping feeding practices.

#### 3.2.4 Latent-indicator standardized indirect effects.

The results in Table 8 highlight the indirect effects of key latent constructs on the three main child growth indicators—wasting, stunting, and MUAC—demonstrating how various determinants influence growth outcomes through intermediary pathways.

**Table 8:** Standardized indirect effects on child growth indicators.

Causal Variable	Effect variable < --	Indirect Effect
**Wasting**	SES	.274
	Food Availability	.552
	Macronutrients	1.441
	Micronutrients	.994
	Neonatal Health	.362
	Feeding Practices	.816
**Stunting**	SES	.109
	Food Availability	.219
	Macronutrients	.573
	Micronutrients	.395
	Neonatal Health	.144
	Feeding Practices	.324
**MUAC**	SES	.121
	Food Availability	.244
	Macronutrients	.636
	Micronutrients	.439
	Neonatal Health	.160
	Feeding Practices	.360

Macronutrient intake shows the strongest indirect effects across all three indicators, particularly for wasting (β = 1.441), followed by MUAC (β = 0.636) and stunting (β = 0.573). This emphasizes the central role of adequate energy and protein intake in preventing both acute and chronic forms of undernutrition. Moreover, Micronutrient intake also demonstrates substantial indirect influence, especially on wasting (β = 0.994), indicating that deficiencies in vitamins and minerals contribute meaningfully to poor nutritional status. Its effects on MUAC (β = 0.439) and stunting (β = 0.395).

Additionally, feeding practices contribute considerable indirect effects on all outcomes, with the strongest effect observed on wasting (β = 0.816), followed by MUAC (β = 0.360) and stunting (β = 0.324). These results show that caregivers’ attitudes, knowledge, and behaviors regarding infant feeding have a meaningful impact on growth, especially in relation to acute malnutrition. Food availability also plays a significant mediating role, with a notable effect on wasting (β = 0.552) and smaller but meaningful effects on MUAC (β = 0.244) and stunting (β = 0.219). This highlights how structural food access challenges, such as issues with food diversity and frequency, contribute indirectly to undernutrition.

Neonatal health, which includes birth weight and gestational age, shows moderate indirect effects across the board, particularly for wasting (β = 0.362), reinforcing the idea that early-life health conditions continue to influence growth outcomes beyond infancy. Socioeconomic status (SES) exerts indirect effects through its impact on dietary intake and food availability. These effects are strongest for wasting (β = 0.274), with smaller but still relevant impacts on MUAC (β = 0.121) and stunting (β = 0.109).

#### 3.2.5 Inter-construct covariances.

The significant covariances presented in Table 9 shows meaningful interrelationships between key constructs and observed variables within the structural model of child growth.

**Table 9:** Significant standardized covariances in the structural equation model.

Causal Variable	Effect variable < -->	Total Effect	S.E.	Significance (P-Value)
Macronutrients	Micronutrients	.816	.008	.003
Food Availability	Feeding Practices	.476	.004	.043
Iron	Folate	.253	.011	<.001
	Calcium	.155	.011	.007

A strong and statistically significant covariance was observed between macronutrient and micronutrient intake (r = 0.816, *p* = .003), indicating that children with higher intake of macronutrients (such as proteins, carbohydrates, and fats) are also more likely to have adequate intake of essential vitamins and minerals. This indicates that dietary patterns tend to be consistently sufficient or insufficient across both macro- and micronutrient dimensions, reinforcing the importance of overall dietary quality rather than isolated nutrient interventions.

The covariance between food availability and feeding practices (r = 0.476, *p* = .043) was also significant, suggesting that households with more reliable access to food tend to engage in more appropriate and informed infant and child feeding behaviors. This relationship indicates how structural access to food resources can influence caregiver decisions, timing, and attitudes toward child nutrition.

At the nutrient level, significant positive covariances were found between iron and folate (r = 0.253, *p* < .001) and between iron and calcium (r = 0.155, *p* = .007). These associations likely reflect shared food sources or dietary patterns where these nutrients co-occur, such as in animal-based or fortified foods. The findings also indicate that deficiencies in one nutrient may coincide with deficiencies in others, demonstrating the importance of broad-spectrum nutritional assessments rather than focusing on single nutrients.

## 4. Discussion

This study utilized a Structural Equation Modeling (SEM) framework to identify the direct and indirect determinants of undernutrition among Palestinian children aged 6–59 months. The findings showed substantial disparities in child nutritional outcomes, driven by geographic, socioeconomic, behavioral, and age-related factors. These patterns reflect a broader context of continuing instability, food insecurity, and limited health infrastructure characteristic of conflict-affected settings.

Children residing in refugee camps were consistently at a nutritional disadvantage compared to those living in villages and urban areas. These findings align with evidence from other protracted humanitarian crises, such as in Lebanon and Syria, where refugee populations face systemic barriers including overcrowding, disrupted health services, and dependency on food assistance [29]. The nutritional vulnerability in these settings stems not only from limited food access but also from a lack of dietary diversity and constrained maternal care. Contrary to conventional economic assumptions, household income did not show a clear protective effect against undernutrition in this context. This pattern reflects the limitations of monetary income as a proxy for food security in regions where economic and political volatility undermines purchasing power and market access. Comparable findings in West Bank and Gaza, as well as in similar conflict-affected contexts like Yemen and South Sudan, emphasize that income-based interventions alone may be insufficient [29–31].

Maternal education, however, showed a much clearer and consistent relationship with child nutrition. Caregivers with higher education levels were more likely to have children with healthier growth patterns. This reinforces extensive literature showing that maternal knowledge significantly influences feeding practices, timely healthcare use, and understanding of dietary needs during critical growth stages [32]. Family size was another influential factor. Larger households tended to report poorer nutritional outcomes, likely due to resource dilution. As the number of household members increases, the ability to provide individualized care and adequate food for each child often diminishes. This has been consistently reported in global studies, especially in South Asian and sub-Saharan African countries, where larger family units are associated with lower per-child food quality and frequency of feeding [33,34].

Widespread deficiencies were found in several key micronutrients, particularly vitamins A, B1, B3, and folate. Notably, older preschool children showed greater deficiencies than younger ones. This is unusual, as younger children are typically considered more vulnerable due to higher nutritional demands. However, this finding likely reflects diminishing access to fortified foods and supplements as children age, and a growing reliance on adult family diets that lack nutrient density. Studies from Guatemala and Bangladesh have similarly noted that preschoolers often “age out” of nutritional safety nets and face increasing risk of deficiencies [35,36].

Despite relatively better adequacy of vitamins B2, B6, and B12, certain groups, such as infants and older preschoolers, still showed signs of marginal intake. This variability highlights the inconsistency in dietary patterns across age groups and suggests a need for sustained nutritional support beyond infancy. Vitamin C and B12 showed moderately adequate intake overall, but deficiencies in infants raise concerns about complementary feeding quality. These early deficits may be due to a limited introduction of fruits, vegetables, and animal-source foods, often replaced by cereal-based diets that do not meet the micronutrient needs of growing children [37].

The results also indicated significant mineral deficiencies, especially for calcium and iron—two nutrients central to bone growth and cognitive development. Calcium intake was particularly poor among older children, which may be attributed to declining consumption of dairy products due to affordability issues or reduced prioritization in household diets. This is consistent with studies from Morocco and Madagascar, where dairy intake is limited and traditional diets fail to meet calcium requirements [38,39]. Iron deficiency remains a major concern, particularly for girls. This sex-based gap may reflect differential access to iron-rich foods, such as meats or fortified cereals. In many parts of the Middle East and North Africa, girls are sometimes deprioritized in food allocation, resulting in higher vulnerability to iron deficiency and anemia [38,39].

Zinc adequacy was somewhat better, though younger and older children still showed signs of insufficiency. Zinc is critical for immune function and growth, and its inadequacy is linked with increased infection susceptibility and stunting [40]. Magnesium and manganese adequacy were generally better, though declining trends in older children again suggest that diet quality does not improve as children transition to adult family meals.

Protein intake was relatively sufficient in older children but inadequate in infants, especially during the weaning period. This reflects common difficulties in achieving protein density in complementary feeding, particularly in households lacking access to affordable animal-source foods. Similar patterns have been observed in East African and South Asian countries, where complementary diets are often plant-based and low in digestible protein [41].

Fat intake was consistently low, especially among the youngest children. Given its essential role in brain development, cellular function, and vitamin absorption, inadequate fat intake during infancy can have long-term developmental implications. These findings align with research that showed lipid-based nutrient supplements significantly improved early growth and micronutrient status [42,43]

Carbohydrate intake improved with age, reflecting greater consumption of main family foods like bread and rice. However, these foods are often energy-dense but low in essential nutrients, leading to diets that are adequate in calories but poor in quality. This trend, commonly reported in urbanizing low-income regions, reflects a shift towards starchy, processed diets that fail to support healthy growth [44].

The structural equation modeling (SEM) results presented in this study demonstrated acceptable-to-excellent fit across key indices, confirming the theoretical plausibility and empirical robustness of the hypothesized pathways. These findings verify the interrelationship of socioeconomic, behavioral, dietary, and early-life health factors in shaping nutritional outcomes, particularly within conflict-affected and resource-constrained settings. The goodness-of-fit statistics indicate that the SEM model adequately represents the underlying data. Low error levels and high levels of precision suggest that the model’s structure reliably captures the relationships between latent constructs and observed outcomes. This aligns with best practices in nutrition research that call for integrating multiple dimensions—social, biological, and behavioral—into a unified analytical framework [26,45]. The confirmation of wasting, stunting, and MUAC as valid indicators of the latent child growth construct supports global anthropometric standards (WHO, 2006), reaffirming their continued utility in assessing both acute and chronic malnutrition.

Among the key determinants, macronutrient intake emerged as the most influential direct predictor of child growth. This is consistent with global evidence highlighting the foundational role of energy-providing nutrients in supporting linear growth, tissue repair, and weight maintenance [46,47]. Protein and fat, in particular, are essential for muscle development and neurocognitive outcomes during early childhood [48].

Socioeconomic status (SES) also demonstrated a statistically significant positive direct effect on growth. This supports earlier findings from both low- and middle-income countries (LMICs) where household income, parental education, and family structure influence food security, healthcare access, and overall child well-being [32,33]. Interestingly, SES also negatively predicted macronutrient intake, showing that families with greater resources might not automatically translate that advantage into better nutrient intake—a pattern also noted in regions with political or logistical barriers to food access, such as Gaza and rural Afghanistan [39]. Neonatal health, captured through gestational age and birth weight, significantly contributed to growth outcomes, reinforcing the importance of prenatal care and maternal nutrition. This is consistent with global studies which link low birth weight to increased risk of stunting and developmental delay [45,49,50]. While micronutrient intake and feeding practices exhibited strong positive effects, their lack of statistical significance may come from variability in caregiver behavior or the nature of nutrient absorption and utilization.

The analysis of indirect effects helps explain how different underlying factors influence child growth. Macronutrient intake stood out as one of the most important factors, especially in reducing cases of wasting. This highlights its key role in preventing short-term or acute malnutrition. Similar patterns have been found in countries like Ethiopia and Bangladesh, where a lack of energy-rich foods is closely linked to poor weight gain in children [34]. Micronutrient intake also had a strong indirect impact, particularly on wasting, pointing to its importance in supporting the immune system, cell development, and recovery from illness [51]. Even though its direct effect wasn’t statistically strong, its indirect role through other factors supports the need for nutrition programs that include both energy and micronutrient-rich foods.

Feeding practices had meaningful indirect effects, especially on wasting and MUAC. These findings support literature from South Asia and Latin America suggesting that caregiver behavior—particularly timing of complementary feeding and food diversity—can significantly alter a child’s nutritional status [29,52]. Importantly, food availability also showed significant mediated effects, indicating that structural barriers such as market access and food prices can indirectly affect growth through dietary quality. Furthermore, the neonatal health contributed moderate indirect effects, particularly on wasting, reaffirming that prenatal and perinatal conditions have lasting consequences on physical growth. Similarly, SES showed indirect influence via dietary and behavioral pathways. These mediated effects illustrate that improvements in income or education must be supported by knowledge translation and access to quality foods to achieve meaningful gains in child nutrition.

The significant covariance between macronutrient and micronutrient intake highlights the interconnectedness of dietary quality. This relationship reflects the broader concept of dietary diversity, where children with access to protein-rich, plant- and animal-based foods are more likely to meet both energy and micronutrient needs [53]. This finding is consistent with dietary pattern research from other studies, where nutrient-rich diets were clustered among households with greater food security and maternal education [14,54,55]

The association between food availability and feeding practices further supports the idea that behavioral interventions cannot succeed without ensuring physical access to food. Caregivers may possess adequate knowledge but remain constrained by food insecurity—a pattern found in humanitarian contexts such as Lebanon [29]. These findings advocate for integrated programs that combine nutrition education with food access initiatives.

The observed covariances between nutrients—such as iron and folate, and iron and calcium—indicate shared food sources and reinforce the need for food-based strategies rather than single-nutrient supplementation. These patterns are biologically plausible, as iron and folate often co-exist in meats and legumes, while calcium and iron frequently appear in dairy and fortified products [35,56,57]

## 5. Conclusion

This study identified key factors influencing child growth among Palestinian children aged 6–59 months using a Structural Equation Modeling approach. Results showed that macronutrient intake, socioeconomic conditions, neonatal health, and infant feeding practices directly impact growth, while micronutrient intake and food availability play important indirect roles. The findings showed that child undernutrition is driven not just by poverty or food shortage but by a combination of diet quality, early health conditions, and caregiver behavior. Improving child nutrition, therefore, requires integrated interventions that enhance feeding knowledge, ensure access to diverse and nutrient-rich foods, and support maternal and neonatal health. This evidence supports multi-sectoral policy responses targeting both the immediate and structural causes of undernutrition in conflict-affected settings like Palestine.

## 6. Limitations

While this study extends crucial insights into child undernutrition in the West Bank, a few key limitations should be noted. First, the cross-sectional design limits our ability to infer causality between variables—that is, while SEM identifies pathways, it does not establish temporal relationships. Second, collected dietary data are subject to recall bias and may not fully capture usual intake, especially for nutrients with seasonal variability. Even with a six-month data collection period, we may have overlooked subtleties of seasonal variation in dietary patterns. Last, some latent constructs, such as socioeconomic status and food availability, were built upon proxy indicators, which may not fully capture the complexity of these concepts. Nevertheless, this study provides a timely and valuable framework for understanding the complex drivers of child nutrition and informing targeted public health interventions.
